# On the Publication of the Results of X-Ray Crystal Structure Analysis in *Metal-Based Drugs*

**DOI:** 10.1155/MBD.2000.i

**Published:** 2000

**Authors:** Edward R. T. Tiekink

**Affiliations:** Department of Chemistry The University of Adelaide 5005 Australia


### EDITORIAL
### On the publication of the results of X-ray crystal structure analysis in *Metal-Based Drugs*

The importance of single crystal structure analysis employing X-ray diffraction methods is undisputed in chemistry/biochemistry. With the advent of area-detector diffractometers and increased automation, it may be anticipated that more structures will be determined and published than previously. The purpose of this Editorial is to set some general guidelines for the publication of crystallographic results in *Metal-Based Drugs*.

The guidelines annunciated below are not meant to circumvent the idea that in a paper 'sufficient detail should be presented in order to allow others to repeat the experiment'. Rather, the guidelines are specified in recognition that many X-ray crystal structure determinations are 'routine' and thereby require no particular or special description of experimental procedures. Authors of papers containing the results of crystal structure determinations are encouraged to check carefully that their submissions match the guidelines below. Of course, deviations from these guidelines will be allowed as long as a justification for the deviation is given.

**Abstract:** While it would be normal to mention that a crystal structure determination is reported, it is not necessary to include crystallographic data in the Abstract.

**Experimental:** For many papers, the Experimental section will comprise a brief statement on how the crystals were obtained and include a suitably modified Table based on the following template:

**Table 1.** Crystal data for

| | | |
|---|---|---|
| Formula | | Formula weight |
| Crystal system | | Crystal size, mm |
| Space group | | *a*, Å |
| *b*, Å | | *c*, Å |
| *β*, ° | | *V*, $Å^3$ |
| *Z* | | Diffractometer |
| Temperature, K | | μ(radiation used), $cm^{-1}$ |
| Trans. factors | | $D_{calcd}$, g $cm^{-3}$ |
| *F*(000) | | $\theta_{max}$, ° |
| Reflns meas. | | Reflns unique, $R_{int}$ |
| Reflns with $I \geq n\sigma(I)$ | | Weighting scheme |
| $R(F \text{ or } F^2)$, $R_w(F \text{ or } F^2)$ | | ρ, e $Å^{-3}$ |
| Programs used | | |
| Deposition number | CCDC XXXXXX | |

This Table reports the key experimental details for a crystal structure analysis and is sufficient for publication of a structure in *Metal-Based Drugs*. Reformatting of the Table to accommodate papers reporting more than one structure is preferable to the inclusion of two (or more) separate Tables. Additional text should be included in the Experimental section when necessary. The additional text could be a discussion of special procedures employed in the data collection and/or refinement, treatment of disorder, location of large residual electron density peaks, details of absolute structure determination, *etc.*

Each structure submitted to *Metal-Based Drugs* **must** have its crystallographic data (preferably in CIF format) deposited at the Cambridge Crystallographic Data Centre and a CCDC deposition number **must** be included in the Table. **Supplementary Material (preferably in CIF format) should still be submitted with manuscripts for the purposes of review.** As the CCDC makes available crystallographic data upon request [1], there is no need in normal circumstances to publish fractional atomic coordinates.

**Discussion:** The description of the structure should include a discussion of key and relevant features of the molecular structure, and the relationship between the structure and other data such as spectroscopic and biological results. It would be appropriate to place the structure in the context of related studies.

A listing of salient interatomic parameters (*e.g.* phenyl C-C distances are not usually required) should be included in tabular form or, in the case of limited data, in the caption to the Figure.

Each structure should be represented by a thermal ellipsoid plot and the probability level for the displacement ellipsoids stated.

When appropriate, an analysis of intermolecular interactions should be included and in these circumstances a unit cell diagram can be published if this enhances the description of the crystal structure.

[1] Copies of data deposited at the CCDC may be obtained free of charge from The Director, CCDC, 12 Union Road, Cambridge CB2 1EZ, England (Facsimile: 44-1223-336033 or e-mail: deposit@ccdc.cam.ac.uk).

On behalf of the Editors, *Metal-Based Drugs*

Edward R. T. Tiekink
Department of Chemistry,
The University of Adelaide,
Australia 5005
Fax: + 61 8 8303 4358
<edward.tiekink@adelaide.edu.au>

